# A Model of Genome Size Evolution for Prokaryotes in Stable and Fluctuating Environments

**DOI:** 10.1093/gbe/evv148

**Published:** 2015-08-04

**Authors:** Piotr Bentkowski, Cock Van Oosterhout, Thomas Mock

**Affiliations:** ^1^School of Environmental Sciences, University of East Anglia, Norwich Research Park, Norwich, United Kingdom; ^2^ Present address: Institute of Environmental Biology, Faculty of Biology, Adam Mickiewicz University, Poznań, Poland

**Keywords:** individual-based model, prokaryotic genome, extinction, evolvability, global change, genome size optimization

## Abstract

Temporal variability in ecosystems significantly impacts species diversity and ecosystem productivity and therefore the evolution of organisms. Different levels of environmental perturbations such as seasonal fluctuations, natural disasters, and global change have different impacts on organisms and therefore their ability to acclimatize and adapt. Thus, to understand how organisms evolve under different perturbations is a key for predicting how environmental change will impact species diversity and ecosystem productivity. Here, we developed a computer simulation utilizing the individual-based model approach to investigate genome size evolution of a haploid, clonal and free-living prokaryotic population across different levels of environmental perturbations. Our results show that a greater variability of the environment resulted in genomes with a larger number of genes. Environmental perturbations were more effectively buffered by populations of individuals with relatively large genomes. Unpredictable changes of the environment led to a series of population bottlenecks followed by adaptive radiations. Our model shows that the evolution of genome size is indirectly driven by the temporal variability of the environment. This complements the effects of natural selection directly acting on genome optimization. Furthermore, species that have evolved in relatively stable environments may face the greatest risk of extinction under global change as genome streamlining genetically constrains their ability to acclimatize to the new environmental conditions, unless mechanisms of genetic diversification such as horizontal gene transfer will enrich their gene pool and therefore their potential to adapt.

## Introduction

The impact of temporal environmental variability on the process of evolution has been an important issue in contemporary ecology and genetics (Kashtan et al. 2007, 2009; Crombach and Hogeweg 2008). As organisms adapt to environmental variability by changing their genes and genomes, it is critical to understand how environmental change impacts genome evolution. Hutchinson addressed the importance of temporal variability of the environment on the diversity of species by studying phytoplankton diversity in relation to resource availability. His early work led to the concept of the “paradox of the plankton” (Hutchinson 1961). This paradox is based on the observation that many species of phytoplankton with similar resource requirements (e.g., nitrate, phosphate) can coexist in the same isotropic and apparently unstructured environment (Hutchinson 1961; Huisman and Weissing 1999) although they are all competing for the same resources. Based on the principle of competitive exclusion (Gause 1932; Szabó and Meszéna 2006), at complete equilibrium the community of phytoplankton species with similar requirements would be reduced to a single species that is optimally adapted to the given conditions. However, Hutchinson (1961) solved the paradox arguing that the equilibrium will never be reached in nature due to temporal variability. Environmental variability is also expected to impact genome evolution, and that natural variation in genome size between organisms may reflect adaptations to different levels of environmental variability.

One of the key elements of genome evolution is related to the evolution of genetic networks, which enable a regulatory response to cope with environmental change (Alon 2007). The interactions between genes in these networks can have many different characters: Genes may depend on common regulatory factors, regulate each other’s expression, encode different peptides of the same enzyme, or different enzymes of the same metabolic pathway. Genetic networks can be divided into discreet functional units in some circumstances facilitating network effectiveness. Here, we will refer to this phenomenon as modularity (Wagner 1996). Parter et al. (2007) classified 117 bacterial species according to the degree of variability in their natural habitat. This study suggested that the modularity of the metabolic network increased with increasing environmental variability. Similarly, also the number of transcription factors and the number of nodes in the metabolic network increased with increasing environmental variation. Genome size was less well correlated with modularity but with phylogenetic distance (Parter et al. 2007). Higher genome network modularity increases a cell’s potential to tackle changing environmental condition and enables it to evolve faster. Genetic regulatory networks are complex and not fully known; hence, Boolean logic circuits are used to approximate certain properties of the real genetic networks (Kauffman 1969; Wynn et al. 2012). In these simplified systems, only off/on states of a gene are permitted without dwelling on parameterization of the expression levels. The control of the regulatory cascades of genes is performed with logic gates known from computer science. A theoretical study of Boolean logic circuits with evolving architecture showed that forcing extinctions in a heterogeneous environment triggered an increased modularity of circuits, whereas the modular architecture did not develop in a homogeneous environment (Kashtan et al. 2009). Also, circuits evolving in variable environments have beneficial mutations arising more frequently than populations from a stable environment. These mutations occur in hubs of the networks, or are directly influencing the hubs, allowing for network-wide rearrangements with minimized deleterious effects (Crombach and Hogeweg 2008).

However, the results by Parter et al. (2007) and Kashtan et al. (2009) can be explained also by nonadaptive processes, such as genetic drift and genetic draft, both of which can shape the architecture of prokaryotic genomes in a nonselective manner (Lynch 2007; Koonin 2011). Prokaryotes are under constant pressure to perfect their metabolic efficiency (Lane and Martin 2010). The ultimate reason for higher modularity of metabolic networks in variable environments is that before the fitness optimum has been reached, the environmental conditions affecting the fitness landscape will have changed again. In other words, in a fluctuating environment, the adaptive fitness-landscape is dynamic and an ever-changing property, which results in continuously changing directions of selection pressure and prevents the species from achieving its maximum metabolic efficiency in any particular environment. The everlasting disequilibrium state might be ecologically more relevant than the theoretical equilibrium states, and as such, transient dynamics are likely to play an important role in ecology and genome evolution (Hastings and Higgins 1994; Hastings 2004; Olszewski 2011). This insight is broadly similar to that of Hutchinson (1961), which allowed to solve the “plankton paradox” (Huisman and Weissing 1999), and which we will explore in this study in more detail using computer simulations.

Here, we developed a population genomics model to investigate genome size evolution in prokaryotes with the focus on how gene number is impacted by resource limitation in both stable and variable environments. Populations of prokaryotic cells feeding on an abstract resource were allowed to evolve in a well-mixed environment, adapting to a single abiotic factor with a given amount of environmental variability denoted by ‘*T*’ (turbulence). Our model assumed a rising regulation burden with an increased number of metabolic genes, and that evolutionary novelty arose only by mutations. Novel genes were acquired through neofunctionalization after gene duplication. Metabolic genes were represented by their phenotypic effect and were responsible for the efficiency of resource uptake under specific abiotic conditions. The efficiency of resource uptake and the costs of gene regulation ultimately determined the fitness of each cell. We furthermore assumed that these metabolic genes were regulated by regulatory genes that carried a fitness cost. Assuming *n* metabolic genes, the fitness cost of regulatory genes is proportional to *n*^2^. With an increasing bacterial genome size, the number of regulatory genes (e.g., *transcription factors*) scales with the power of approximately 2 when the number of metabolic genes grows linearly (Van Nimwegen 2003; Molina and van Nimwegen 2008, 2009; Koonin 2011). Finally, given that noncoding DNA tends to account for less than 15% of the total DNA of prokaryotes (Mira et al. 2001; Lynch 2006), we ignored noncoding DNA in our model. To keep our model simple, we have neither included horizontal gene transfer (HGT) nor genomic islands which, however, are important mechanisms of genome diversification (Coleman et al. 2006; Isambert and Stein 2009; Avrani et al. 2011; Koonin 2011; Fernández-Gómez et al. 2012). The output of the model included the number of genes and their individual contribution to fitness were both physical properties (trait values) resulting from adaptive evolution. In addition, we examined the evolvability of populations in both stable and fluctuating environments expressed as their ability to adapt and resist extinction.

## Materials and Methods

### Model

The model represents a resource-limited population of free-living haploid prokaryotic cells. Each cell is an independent agent (Grimm 1999) that can take up resources and has a cost of living. A cell can die because of starvation or random causes. The fitness of each cell depends on the match between the environmental value to which its genes are adapted and its environment. The fitness of a cell determines the amount of resources it can acquire (or lose) in each time step. Depending on its fitness value, a cell either can continue to live and acquire (or lose) resources, or it can die. Once a cell has acquired sufficient resources, it is able to reproduce asexually through clonal reproduction and produce offspring that are identical to its own genotype baring mutations.

### Environment

The state of the environmental condition is represented by only one single variable *x* which is a real number with a range between [−1, +1]. The boundaries of *x* are limited as boundaries of many abiotic conditions can also be considered finite, such as, for example, pH. Other, similar types of environmental variables are temperature, irradiance, and nutrients. These types of abiotic variables have one particular value at a time, but the range and rate of their change in time define the environment. In our model, the state of environmental conditions changes in time: *x*(*t*). Mode of change of *x*(*t*)*→x*(*t + *1) is dictated by a bounded random walk within the interval [−1, +1]:

(1) $$x\left(t_{0}\right)=0\wedge x\left(t+1\right)=x\left(t\right)+RND\left[-T,T\right]\wedge \left|x\left(t+1\right)\right|\leq 1,$$

where *RND*[*−T, T*] randomly generates a real number from the interval [*−T, T*]. Random walk allows to control the rate of change without the need to control other factors, for example, periodicity of a more regular function. The value of *T* varies between 0 and 0.5 and it is the turbulence level which controls the variability of the environment. A large value of *T* represents an unpredictable/variable environment. Also, the environment has a constant pool of resources that represent available nutrients (*R*). A fraction of *R* is allocated to cells, whereas the remainder represents the pool of free resources. Cells can utilize these free resources for growth and reproduction, they return all their accumulated resources when they die and a portion in each time step to account for living expenses.

### Genes and Genotypes

**Fig. 1.—:**
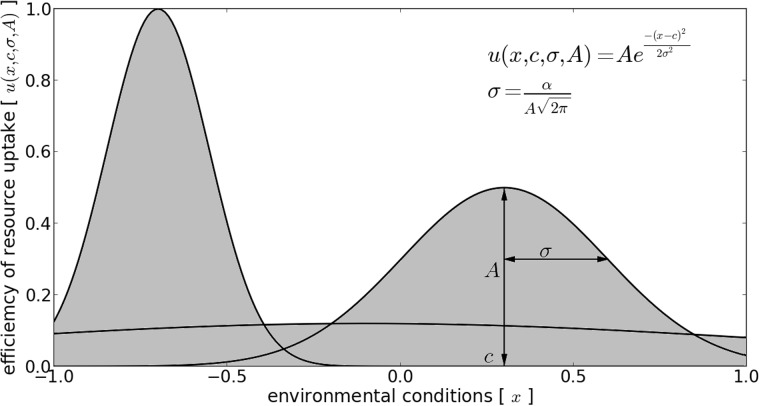
A genotype consisting of three genes in the environmental condition space. Surfaces under all the Gaussian curves are equal and scaled by factor *α* (eq. 3). The total shaded area represents the uptake efficiency function *U*(*x*) of this particular genotype.

Genes are represented directly by their resource uptake function, that is, the function that describes the efficiency with which a gene can uptake resources in different values of the environment *x*. The efficiency of resource uptake is given by a Gaussian function:

(2) $$u\left(x,c,\sigma ,A\right)=A\cdot \text{exp}\left(\frac{-{\left(x-c\right)}^{2}}{2\sigma^{2}}\right),$$

where *x* is the space of environmental conditions within the interval [−1, +1], *c* is the location of the maximum value of *u* (*x, c, σ, A*) in the space of environmental conditions (*c* ∈ [−1, +1]), and *A* is the maximum value of *u* (*A* ∈ [0, 1]) which represents the maximum efficiency of resource uptake of a gene. Parameter *σ* is the dispersion controlling the width of the Gaussian curve and it is obtained from the following equation:

(3) $$\sigma =\frac{\alpha }{A\sqrt{2\pi }},$$

where *α* is a constant factor which scales the surface under the Gaussian curve allowing to control the fraction of the total environmental space one single gene can cover. Figure 1 shows an example of a genotype. Note that setting this surface as fixed entity adds a constrained for *σ* being dependent on *A* (eq. 3). If the cell *i* has more than one gene, the value of efficiency of resource uptake *U_i_* (*x*) for a given value of *x* is taken from the gene that has the highest value for that *x* (see shaded area on fig. 1):

(4) $$U_{i}\left(x\right)=\text{MAX}\left(u_{1}\left(x\right),u_{2}\left(x\right),...,u_{n}\left(x\right)\right).$$

A cell can have *n* number of genes that generate a cost growing with gnome size as:

(5) $$K=\gamma \left(n+n^{2}\right)+\kappa ,$$

where *κ* is a constant “cost of living” and *γ* is a scaling factor for gene-associated costs. This is dictated by the “genome scaling laws,” observations which showed that the number of metabolic genes grows linearly and the number of regulatory genes grows in a square proportion to the genome size (Van Nimwegen 2003; Molina and van Nimwegen 2009; Koonin 2011).

### Life Cycle of Cells

The census population size (i.e., the total number of cells in the population) is determined by the amount of total resources in the environment *R_env_*. In each time step, a cell *i* can either return to the population *K_i_* (eq. 5) of its internal resource, or it is randomly selected to die with fixed probability *δ*. The uptake of resources *U_i_*(*x*) (eq. 4) of the *i*th cell depends on the availability of the free resource and the cell’s genotype. Cells are picked one-by-one at random and allocated the amount of resource according to their *U_i_*(*x*) value for current value of *x*(*t*). When the free resource in depleted, the feeding procedure is terminated and the model moves to the next step leaving the remaining cells in the queue unfed. If a cell’s internal resource falls below a minimum threshold (*r_min_*) the cell dies, and its internal resources are reallocated to the free pool. When the resources gathered by the cell exceed *r_rep_*, the cell reproduces clonally, and it reallocates half of its resources to each copy. At the reproduction, three kinds of mutations can happen to each of two new cells: Deletions (removal of a gene from the cell’s genotype), duplications (duplication of an existing gene in the cell’s genome which results in a cell having two copies of the same gene), and modifications (change of shape of the Gaussian form of an existing gene). All three happen randomly and independently with equal probabilities *µ*. Gene duplication increases the cost of operating a genome, but it does not increase *U_i_*(*x*) given that both orthologous genes will initially have an identical uptake function (see shaded area on fig. 1 and eq. 4) (see the supplementary material and the source code of the simulation program, Supplementary Material online, for further details).

### Statistics

The purpose of the model was to evaluate the evolution of genome size under different environmental scenarios (stable and fluctuating environment). For this purpose, the following statistics have been introduced: 1) The grand mean number of genes, which is the mean number of genes averaged over all cells at the time when gene numbers have stabilized; and (2) the rate of adaptive evolution, which is the mean number of mutations that became fixed in each clonal lineage (see supplementary material, Supplementary Material online, for further details).

## Results

**Fig. 2.—:**
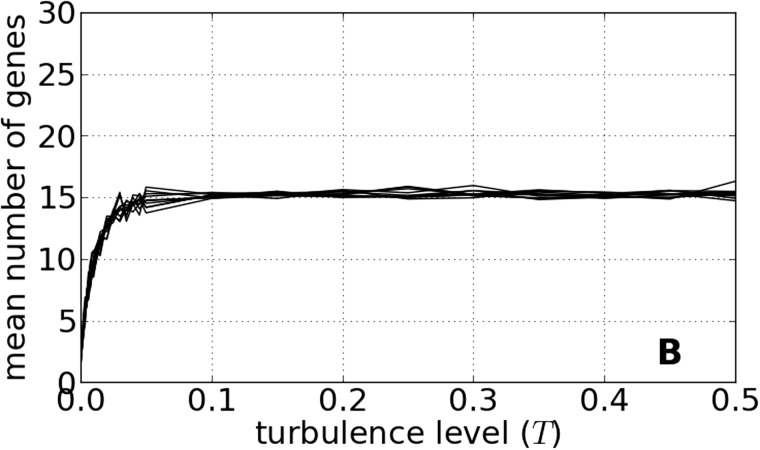
The mean number of genes as the function of the turbulence level *T*. Ten simulations set to the standard parameter values show consistent results regarding the gene number. Uncertainty has been omitted for the sake of simplicity of the graphs. Each series of runs was initialized with a different seed of the pseudorandom number generator.

**Fig. 3.—:**
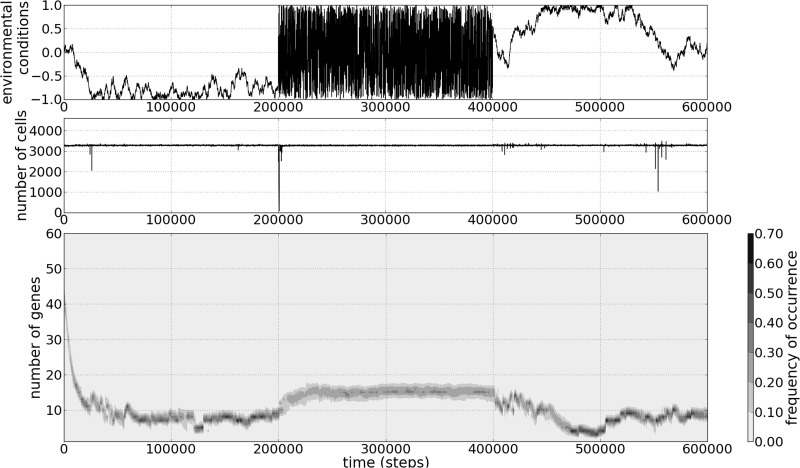
Optimization of genome sizes in an environment with modulated turbulence level. Simulation was initialized with genome ranges of *η_0_* ∈ [40, 60] of genes per genome. Turbulence level was *T* = 0.005 at the beginning, *T* = 0.2 in the middle of simulation, and again *T* = 0.005 at the end (see the upper panel).

Genome size increases with increased level of environmental instability (i.e., turbulence, *T*) (fig. 2). The genome size expands rapidly from two genes in a constant environment (*T* = 0) to around 15–16 genes per genome at *T* = 0.05. A further increase in environmental turbulence has no effect on the number of genes (fig. 2). Furthermore, when faced with changing environmental conditions, the genome size evolves, attaining a gene number that is optimum for the contemporary environment and level of turbulence (fig. 3). Going from an environment with low to high turbulence, the population crashed (fig. 3, after *t* = 2 × 10^5^), whereas the population remained stable when going from high to low turbulence (fig. 3, after *t* = 4 × 10^5^). Unexpectedly, however, if the level of turbulence remained stable over time, the frequency of crashes of simulated populations was highest in environments with low to medium turbulence *T* ∈ [0.005, 0.05], whereas the populations were more stable in constant environments (*T* = 0) and environments with high turbulence *T > *0.1.

**Fig. 4.—:**
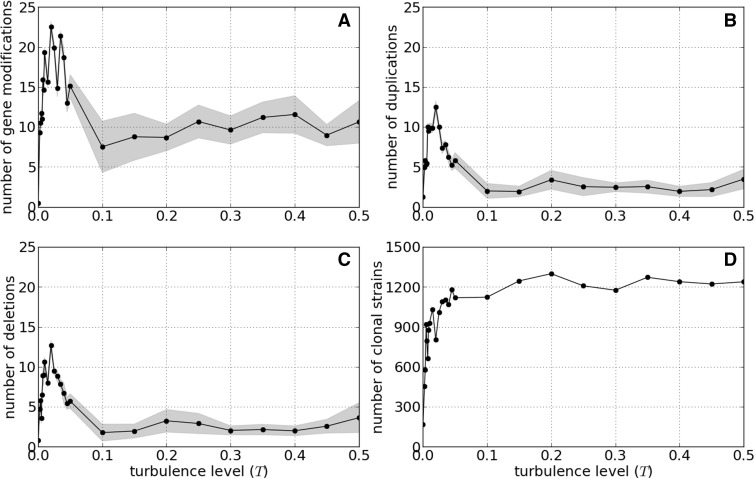
Mean number of mutations per clonal strain as a function of the turbulence level *T*. Each dot represents the mean number of mutations of one of the three kinds (panel *A*—gene modifications, *B*—duplications, and *C*—deletions) calculated for the last 10^5^ time steps (out of total 2 × 10^5^), after the system has stabilized; shaded area is SD, calculated in a similar manner. Panel (*D*) presents the number of clonal strains at the end of the run.

The speed of evolution expressed as the number of fixed mutations per 10^5^ steps of the simulation was lowest in a constant environment. However, the highest rate of evolution was observed in moderately turbulent environments *T* ∈ [0.005, 0.05] and not in highly turbulent environments (fig. 4*A*–*C* ). High level of environmental turbulence resulted in a moderate rate of evolution. Gene modifications (e.g., single nucleotide polymorphisms) are the most frequent type of mutations, and they are about twice as frequent as duplications or deletions.

**Fig. 5.—:**
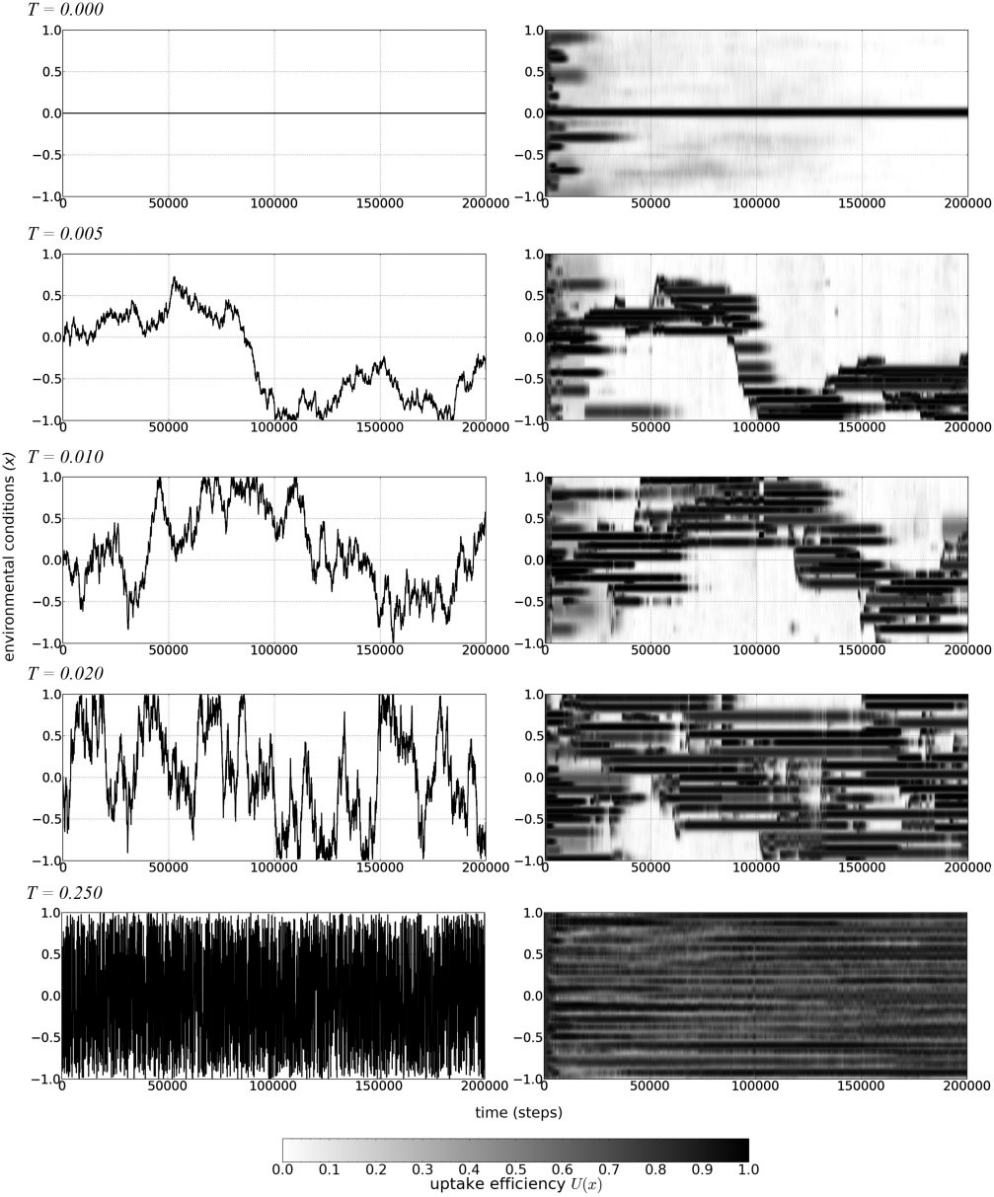
Genome shapes evolve in time and depend on the turbulence level. Each pair of panels represents one simulation with different turbulence level *T* values (number above the panels on the left). Left panels present the change of environmental conditions *x* in time during the whole simulation. Right panels show heat maps of the mean uptake efficiency *U*(*x*) averaged for the whole population evolving in time. The intensity of black is proportional to the value of mean uptake efficiency *U*(*x*) for the given environmental conditions *x* in time step *t* (see the bar below panels). Standard deviation of *U*(*x*) has been omitted due to the clarity of the presentation.

Finally, a high rate of evolution does not appear to be accompanied by the rise in the number of clonal strains (fig. 4*D*). This suggests that the high genomic diversity of clones in the high turbulence environment was not the result of an accelerated rate of evolution. Rather, clonal diversity was maintained in the unpredictable environment because of the relatively low extinction risk of lineages, resulting in a phenotypically diverse population. Under these conditions, selection favors the evolution of generalist “multipurpose” genotypes with large genome sizes adapted to a range of environmental conditions (compare upper and lower panels on fig. 5).

## Discussion

We built an individual-based model to simulate genome size evolution (i.e., the number of genes in a genome) in response to environmental perturbations and variation. The model simulated a single environmental parameter that affected the efficiency with which gene products could utilize a limited resource. Individuals could acquire multiple gene copies only through duplication. Each paralogous gene was able to evolve through mutations and adapt to function in a novel environmental space. However, individuals incurred a regulatory cost for each additional gene copy. The population evolved until a dynamic equilibrium was reached in which individuals carried an optimum number of genes, that is, “genome streamlining” (Giovannoni et al. 2005, 2014; Swan et al. 2013). The four principal findings are that 1) genome size increased with increased level of environmental turbulence, 2) the rate of evolution was highest in moderately turbulent environments, (3) clonal diversity was highest in the most turbulent environments, and (4) population extinction rates were highest in populations that had evolved in relatively stable environments.

Computational modelling of evolutionary process has notably advanced in the last two decades (Adami 2006; Hindré et al. 2012; Mozhayskiy and Tagkopoulos 2013; Pigliucci 2013). A number of theoretical publications have shown that varying or unstable environments produce genetic networks that are modular (Kashtan and Alon 2005; Kashtan et al. 2009). Furthermore, unstable conditions influence simulated genetic network architecture, which allows for network-wide rearrangement by altering only a few specific genes (Crombach and Hogeweg 2008). Thus, genomic re-engineering caused by environmental fluctuations might accelerate the rate of evolution (Kashtan et al. 2007). Variations in nutrient supply forced simulated metabolic networks to develop more multifunctional enzymes, making these genotypes more robust to cope with gene deletion (Soyer and Pfeiffer 2010). Correspondingly, opportunistic microbes such as *Escherichia coli* and *Saccharomyces cerevisiae* might lose 37–47% of their metabolic reactions without blocking the production of any biomass component under any nutritional conditions (Wang and Zhang 2009). This demonstrates the biological significance of redundancy of gene networks. Although *Pelagibacter* (Proteobacteria) and *Prochlorococcus* (Cyanobacteria), two phyla of marine bacteria, are known for extreme genome streamlining (Giovannoni et al. 2005, 2014), their genomes contain islands of genomic variability acquired through HGT (Coleman et al. 2006; Avrani et al. 2011; Grote et al. 2012) giving them metabolic advantage to adapt to local environments and to defend viral attacks (Coleman et al. 2006; Avrani et al. 2011; Grote et al. 2012). Nonetheless, even with the presence of genomic islands, *Pelagibacter ubique* has the smallest genome among free-living bacteria known to date with only obligate parasites or symbionts having smaller ones (Giovannoni et al. 2005).

Our study indicates that a fluctuating environment produces large genomes that are more robust to variable conditions, and consequently, that such populations suffer fewer extinctions. This conclusion is entirely unremarkable until one realizes how this has been accomplished, as well as its wider implications. Rather than evolution directly selecting for genes that encode for a more plastic phenotype, these larger genomes evolve in response to a relaxed selection pressures on metabolic efficiency. Fierce resource competition between genotypes in stable environments results in overly optimized and highly streamlined genomes (Giovannoni et al. 2014). In reference to Hutchinson’s (1961) solution of the plankton paradox, we argue that variation in gene number and genome size reflects adaptations to different levels of environmental variability and nutrient supply (Konstantinidis and Tiedje 2004). Our study furthermore implies that species evolved in relatively stable environments would face the greatest extinction risk in a globally changing environment as the result of genome streamlining unless other mechanisms of gaining and maintaining genetic diversity come into play such as HGT.

## Data Availability

Animation 1: Evolution in low-turbulence environment https://dl.dropboxusercontent.com/u/834906/anim_T_low_0.01.mp4

Animation 2: Evolution in high-turbulence environment https://dl.dropboxusercontent.com/u/834906/anim_T_high_0.25.mp4

Code and documentation of the programme: https://dl.dropboxusercontent.com/u/834906/Bentkowski_et_al_2015_Code.zip

## Supplementary Material

Supplementary material is available at *Genome Biology and Evolution* online (http://www.gbe.oxfordjournals.org/).

Supplementary Data

## References

[evv148-B1] Adami C 2006 Digital genetics: unravelling the genetic basis of evolution. Nat Rev Genet. 7:109–118. 16418746 10.1038/nrg1771

[evv148-B2] Alon U 2007 An introduction to systems biology: design principles of biological circuits. Boca Raton (FL): Chapman & Hall/CRC.

[evv148-B3] Avrani S Wurtzel O Sharon I Sorek R Lindell D 2011 Genomic island variability facilitates *Prochlorococcus*-virus coexistence. Nature 474:604–608. 21720364 10.1038/nature10172

[evv148-B4] Coleman ML 2006 Genomic islands and the ecology and evolution of *Prochlorococcus*. Science 311:1768–1770. 16556843 10.1126/science.1122050

[evv148-B5] Crombach A Hogeweg P 2008 Evolution of evolvability in gene regulatory networks. PLoS Comput Biol. 4:e1000112. 18617989 10.1371/journal.pcbi.1000112 PMC2432032

[evv148-B6] Fernández-Gómez B 2012 Patterns and architecture of genomic islands in marine bacteria. BMC Genomics 13:347. 22839777 10.1186/1471-2164-13-347 PMC3478194

[evv148-B7] Gause GF 1932 Experimental studies on the struggle for existence. J Exp Biol. 9:389–402.

[evv148-B8] Giovannoni SJ 2005 Genome streamlining in a cosmopolitan oceanic bacterium. Science 309:1242–1245. 16109880 10.1126/science.1114057

[evv148-B9] Giovannoni SJ Thrash JC Temperton B 2014 Implications of streamlining theory for microbial ecology. ISME J. 8:1553–1565. 24739623 10.1038/ismej.2014.60 PMC4817614

[evv148-B10] Grimm V 1999 Ten years of individual-based modelling in ecology: what have we learned and what could we learn in the future? Ecol Modell. 115:129–148.

[evv148-B11] Grote J 2012 Streamlining and core genome conservation among highly divergent members of the SAR11 clade. MBio 3:1–13. 10.1128/mBio.00252-12 PMC3448164 22991429

[evv148-B12] Hastings A 2004 Transients: the key to long-term ecological understanding? Trends Ecol Evol.. 19:39–45. 16701224 10.1016/j.tree.2003.09.007

[evv148-B13] Hastings A Higgins K 1994 Persistence of transients in spatially structured ecological models. Science 263:1133–1136. 17831627 10.1126/science.263.5150.1133

[evv148-B14] Hindré T Knibbe C Beslon G Schneider D 2012 New insights into bacterial adaptation through in vivo and in silico experimental evolution. Nat Rev Microbiol. 10:352–365. 22450379 10.1038/nrmicro2750

[evv148-B15] Huisman J Weissing FJ 1999 Biodiversity of plankton by species oscillations and chaos. Nature 402:407–410.

[evv148-B16] Hutchinson GE 1961 The paradox of the plankton. Am Nat. 95:137–145.

[evv148-B17] Isambert H Stein R 2009 On the need for widespread horizontal gene transfers under genome size constraint. Biol Direct. 4:28. 19703318 10.1186/1745-6150-4-28 PMC2740843

[evv148-B18] Kashtan N Alon U 2005 Spontaneous evolution of modularity and network motifs. Proc Natl Acad Sci U S A. 102:13773–13778. 16174729 10.1073/pnas.0503610102 PMC1236541

[evv148-B19] Kashtan N Noor E Alon U 2007 Varying environments can speed up evolution. Proc Natl Acad Sci U S A. 104:13711–13716. 17698964 10.1073/pnas.0611630104 PMC1948871

[evv148-B20] Kashtan N Parter M Dekel E Mayo AE Alon U 2009 Extinctions in heterogeneous environments and the evolution of modularity. Evolution 63:1964–1975. 19473401 10.1111/j.1558-5646.2009.00684.x PMC2776924

[evv148-B21] Kauffman S 1969 Homeostasis and differentiation in random genetic control networks. Nature 224:177–178. 5343519 10.1038/224177a0

[evv148-B22] Konstantinidis KT Tiedje JM 2004 Trends between gene content and genome size in prokaryotic species with larger genomes. Proc Natl Acad Sci U S A. 101:3160–3165. 14973198 10.1073/pnas.0308653100 PMC365760

[evv148-B23] Koonin EV. 2011 The logic of chance: the nature and origin of biological evolution. Upper Saddle River (NJ): FT Press.

[evv148-B24] Lane N Martin W 2010 The energetics of genome complexity. Nature 467:929–934. 20962839 10.1038/nature09486

[evv148-B25] Lynch M 2006 Streamlining and simplification of microbial genome architecture. Annu Rev Microbiol. 60:327–349. 16824010 10.1146/annurev.micro.60.080805.142300

[evv148-B26] Lynch M. 2007 The origins of genome architecture. Sunderland (MA): Sinauer Associates Inc.

[evv148-B27] Mira A Ochman H Moran NA 2001 Deletional bias and the evolution of bacterial genomes. Trends Genet. 17:589–596. 11585665 10.1016/s0168-9525(01)02447-7

[evv148-B28] Molina N van Nimwegen E 2008 The evolution of domain-content in bacterial genomes. Biol Direct. 3:51. 19077245 10.1186/1745-6150-3-51 PMC2615428

[evv148-B29] Molina N van Nimwegen E 2009 Scaling laws in functional genome content across prokaryotic clades and lifestyles. Trends Genet. 25:243–247. 19457568 10.1016/j.tig.2009.04.004

[evv148-B30] Mozhayskiy V Tagkopoulos I 2013 Microbial evolution in vivo and in silico: methods and applications. Integr Biol. 5:262–277. 10.1039/c2ib20095c 23096365

[evv148-B31] Olszewski TD 2012 Persistence of high diversity in non-equilibrium ecological communities: implications for modern and fossil ecosystems. Proc R Soc Lond B Biol Sci. 279:230–236. 10.1098/rspb.2011.0936 PMC3223685 21653592

[evv148-B32] Parter M Kashtan N Alon U 2007 Environmental variability and modularity of bacterial metabolic networks. BMC Evol Biol. 7:1–8. 17888177 10.1186/1471-2148-7-169 PMC2151768

[evv148-B33] Pigliucci M 2013 On the different ways of “doing theory” in biology. Biol Theory. 7:287–297.

[evv148-B34] Soyer OS Pfeiffer T 2010 Evolution under fluctuating environments explains observed robustness in metabolic networks. PLoS Comput Biol. 6:e1000907. 20865149 10.1371/journal.pcbi.1000907 PMC2928748

[evv148-B35] Swan BK 2013 Prevalent genome streamlining and latitudinal divergence of planktonic bacteria in the surface ocean. Proc Natl Acad Sci U S A. 110:11463–11468. 23801761 10.1073/pnas.1304246110 PMC3710821

[evv148-B36] Szabó P Meszéna G 2006 Limiting similarity revisited. Oikos 112:612–619.

[evv148-B37] Van Nimwegen E 2003 Scaling laws in the functional content of genomes. Trends Genet. 19:479–484. 12957540 10.1016/S0168-9525(03)00203-8

[evv148-B38] Wagner GP 1996 Homologues, natural kinds and the evolution of modularity. Am Zool. 36:36–43.

[evv148-B39] Wang Z Zhang J 2009 Abundant indispensable redundancies in cellular metabolic networks. Genome Biol Evol. 1:23–33. 20333174 10.1093/gbe/evp002 PMC2817398

[evv148-B40] Wynn ML Consul N Merajver SD Schnell S 2012 Logic-based models in systems biology: a predictive and parameter-free network analysis method. Integr Biol. 4:1323–1337 10.1039/c2ib20193c PMC3612358 23072820

