# Supplementary material for: A Model of Genome Size Evolution for Prokaryotes in Stable and Fluctuating Environments
Source: Genome Biol Evol. 2015 Aug 4;7(8):2344–51. doi: 10.1093/gbe/evv148 (PMC4558865; doi:10.1093/gbe/evv148)
Supplement: Supplementary Data [file supp_7_8_2344__index.html]

Supplementary Data 

# A model of genome size evolution for prokaryotes in stable and fluctuating environments

## Supplementary Data

files

- Supplementary Data - docx file
