# Supplementary material for: A Model of Genome Size Evolution for Prokaryotes in Stable and Fluctuating Environments
Source: Genome Biol Evol. 2015 Aug 4;7(8):2344–51. doi: 10.1093/gbe/evv148 (PMC4558865; doi:10.1093/gbe/evv148)
Supplement: Supplementary file 2 [file supp_evv148_supplementary.docx]

Supplementary Material

Animation 1: Evolution in low-turbulence environment 

[https://dl.dropboxusercontent.com/u/834906/anim_T_low_0.01.mp4](https://dl.dropboxusercontent.com/u/834906/anim_T_low_0.01.mp4)

Animation 2: Evolution in high-turbulence environment

[https://dl.dropboxusercontent.com/u/834906/anim_T_high_0.25.mp4](https://dl.dropboxusercontent.com/u/834906/anim_T_high_0.25.mp4)

Code and documentation of the programme:

[https://dl.dropboxusercontent.com/u/834906/Bentkowski_et_al_2015_Code.zip](https://dl.dropboxusercontent.com/u/834906/Bentkowski_et_al_2015_Code.zip)
